# Deep brain stimulation effects on verbal fluency dissociated by target and active contact location

**DOI:** 10.1002/acn3.51304

**Published:** 2021-02-17

**Authors:** Kevin D. John, Scott A. Wylie, Benoit M. Dawant, William J. Rodriguez, Fenna T. Phibbs, Elise B. Bradley, Joseph S. Neimat, Nelleke C. van Wouwe

**Affiliations:** ^1^ Department of Neurological Surgery University of Louisville Louisville KY USA; ^2^ Department of Electrical Engineering and Computer Science Vanderbilt University Nashville TN USA; ^3^ Department of Neurology Vanderbilt University Medical Center Nashville TN USA

## Abstract

**Objective:**

Deep brain stimulation (DBS) improves motor symptoms in Parkinson’s disease (PD), but it can also disrupt verbal fluency with significant costs to quality of life. The current study investigated how variability of bilateral active electrode coordinates along the superior/inferior, anterior/posterior, and lateral/medial axes in the subthalamic nucleus (STN) or the globus pallidus interna (GPi) contribute to changes in verbal fluency. We predicted that electrode location in the left hemisphere would be linked to changes in fluency, especially in the STN.

**Methods:**

Forty PD participants treated with bilateral DBS targeting STN (*n* = 23) or GPi (*n* = 17) completed verbal fluency testing in their optimally treated state before and after DBS therapy. Normalized atlas coordinates from left and right active electrode positions along superior/inferior, anterior/posterior, and lateral/medial axes were used to predict changes in fluency postoperatively, separately for patients with STN and GPi targets.

**Results:**

Consistent with prior studies, fluency significantly declined pre‐ to postsurgery (in both DBS targets). In STN‐DBS patients, electrode position along the inferior to superior axis in the left STN was a significant predictor of fluency changes; relatively more superior left active electrode was associated with the largest fluency declines in STN. Electrode coordinates in right STN or GPi (left or right) did not predict fluency changes.

**Interpretation:**

We discuss these findings in light of putative mechanisms and potential clinical impact.

## Introduction

Deep brain stimulation (DBS) for the treatment of Parkinson’s disease (PD) is an effective surgery for the reduction of PD motor symptoms but can also have disruptive cognitive side effects such as decline in spontaneous word generation, or verbal fluency.^1^, ^2^, ^3^, ^4^, ^5^, ^6^, ^7^, ^8^, ^9^, ^10^, ^11^, ^12^ Verbal fluency is generally measured by asking patients to verbalize, as quickly as possible, words that are representative of a predefined category. For example, patients might be given 1 min to name as many words as they can from a specific category, such as types of animals or foods found at a grocery store (*semantic* fluency), which is easier than naming words that do not share a clear semantic structure, such as words that begin with a particular letter of the alphabet (often called *phonemic* fluency). Patients with PD also experience selective reductions in the so‐called *action* fluency related to naming words that reflect actions or movements (e.g., running, writing^13^). Deficits in verbal fluency are variably expressed in PD and variably exacerbated following DBS surgery.^14^ Not surprising, reductions in verbal fluency produce negative impact on the quality of life.^15^ The current study contributes to the ongoing efforts to identify factors that predict postoperative fluency decline with DBS.

A recent series of meta‐analytic and qualitative reviews confirm that deficits in verbal fluency represent one of the most reported side effects of DBS therapy when targeting the subthalamic nucleus (STN) or the globus pallidus interna (GPi).^14^, ^16^ However, the reviews also highlight that insights about the expression, scope, and causes of the effect across individuals and between targets are incomplete. Some patients do not show any fluency deficits, whereas others show dramatic reductions in fluency.

The production of speech involves a complex interplay of motor and cognitive processes, and the decline of verbal fluency in PD patients receiving DBS is thought to be caused by changes in the basal‐ganglia‐thalamocortical network.^17^, ^18^ The most promising factors surround the surgical procedure or stimulation parameters.^8^, ^16^, ^19^, ^20^, ^21^ Nonsurgical contributors, such as dopaminergic medication changes, presurgical disease variables, and various neuropsychological and physical characteristics appear to provide minimal predictive insight into the emergence of fluency deficits.^16^

For example, fluency deficits have been reported coincident with the surgical placement of DBS electrodes before the device is turned on, suggesting that either a microlesion effect in the target structure, or disruption to structures outside of the target (e.g., caudate nucleus, anterior cingulate cortex) impacted by the descending electrode may contribute to fluency declines.^16^, ^22^, ^23^, ^24^, ^25^

However, there is also evidence for reductions in fluency tied to clinically relevant high‐frequency DBS (~130 Hz) compared to no stimulation or low‐frequency DBS (~10–60 Hz), implicating a direct contributory role of stimulation.^26^, ^27^, ^28^ Additionally, there is some indication that fluency deficits may be more pronounced with STN relative to GPi stimulation, although mixed results have been reported with both targets.^26^, ^27^, ^28^

A key question arising from stimulation effects on fluency is whether the effect is more pronounced by stimulating either hemisphere or specific subregions within a hemisphere of the target structure (i.e., STN, GPi). In other words, how might the placement of the electrode(s) used to achieve clinical improvement in motor symptoms contribute to the emergence of fluency difficulties. The STN is thought to be comprised of functional subregions, a dorsolateral sensorimotor area, a central associative territory, and medial limbic region, that receive projections from dissociable cortical areas.^29^ The GPi is similarly associated with distinct functional subregions, but is a much larger structure than the STN and the GPi motor region occupies around 50% of its volume.^29^, ^30^, ^31^, ^32^ These characteristics of the GPi may limit the impact of variability in contact location on these functional circuitries, although hemispheric placement may still be a factor. Determining how fluency effects are influenced by electrode positioning within each target and hemisphere may provide important insight regarding functional circuitries and offer new strategies for mitigating negative DBS side effects.

A handful of studies have investigated the role of electrode positioning on fluency effects. Among 31 PD patients with predominantly bilateral STN‐DBS, Witt et al.^33^ reported that patients with fluency declines (>1 SD reduction in fluency) had electrode positions outside of the volume of electrode positions constructed for the stable, or improved, performers (<1 SD reduction in fluency) in the left hemisphere, although the precise coordinates of these electrode position differences within the left STN could not be specified. Okun et al.^30^ studied PD patients treated with either left or right unilateral DBS delivered to STN (*n* = 22) or GPi (*n* = 23) targets. In four counter‐balanced sessions, patients performed verbal fluency measures with and without stimulation delivered to clinically optimal contacts as well as to contacts located dorsal and ventral to the optimal contacts. They reported a global reduction in fluency when stimulating the STN, irrespective of which contact was stimulated, but no measurable fluency reduction when DBS targeted any of the GPi contacts. Per the authors, sample size issues prevented analysis of the contribution of left and right hemispheres to the fluency reductions induced by STN stimulation.

In follow‐up studies with the same cohort of patients with unilateral DBS, Mikos and colleagues further investigated how the modeled volume of tissue activation in the STN^34^ and GPi^35^ at clinically optimal, dorsal to optimal contacts, and ventral to optimal contact stimulation conditions, contributed to fluency effects. Fluency declined when stimulating a larger region contained within the ventral STN,^34^ but no impact of stimulation across contacts within the GPi was found.^35^ Again, investigation of lateralization of effects was precluded by sample size limitations. Finally, in 14 PD patients treated with bilateral STN, Ehlen et al.^36^ reported no global reductions of verbal fluency with STN stimulation. However, stimulation amplitude and posterior electrode placements in the left hemisphere were associated with fluency change, that is, they found larger fluency improvements with more anterior positioned electrodes in the left STN and with larger stimulation amplitudes.

Collectively, the results from these studies are suggestive that STN stimulation effects on fluency may be partly related to the location of electrodes, potentially in the left STN and involving placements along the dorsal–ventral and/or anterior–posterior axes. In contrast, no indications about the role of electrode location in the GPi are currently evident. In the current study, we add to this emerging body of work by measuring fluency changes from pre‐ to post‐DBS in patient who received DBS targeting STN or GPi. Like Witt and Ehlen et al.,^33^, ^36^ we studied patients with (predominantly) bilateral lead placements. We were particularly interested in comparing the relationship between fluency changes and electrode placement coordinates between the left and right hemispheres. We also determined the clinical coordinates along the superior–inferior, lateral–medial, and anterior–posterior axes for the clinically optimal contacts and for each hemisphere. In line with prior indications, we predicted that the location of the electrode in the *left* STN would be associated with changes in verbal fluency as opposed to coordinate locations in the *right* STN placement. A comparison of left versus right hemispheric coordinates in the GPi has not been studied.

We were less committed regarding the prediction of which axis would be most tightly aligned with fluency effects. As noted above, both anterior–posterior and inferior–superior axes of active electrode contacts have been linked to DBS fluency effects.

## Methods

### Participants

Forty PD patient were recruited from the Movement Disorders and Neurosurgical DBS clinics at Vanderbilt University Medical Center. Patients either underwent DBS of the STN (*n* = 23; 21 bilateral and 2 left unilateral) or GPi (*n* = 17; 14 bilateral and 3 left unilateral), using conventional neurosurgical procedures.^37^ Age, disease duration, cognitive status, education, BDI, and medication intake were similar between GPi and STN targets. The selection of target was determined during a case conference of the Vanderbilt Movement Disorders Surgery group. Some of the factors that contributed to selection of the DBS target included: the frequency of a patient’s falls, symptoms of depression, medication response and dosing; baseline verbal fluency (semantic and phonemic); and/or the presence of hypophonia. Generally, patients with low medication requirements, symptoms of depression, frequent falls, and concerns about fluency were more likely to receive GPi surgery.

The exclusion criteria for this study included: MMSE score of less than 25, a history of neurological disorders other than PD, a diagnosis of psychiatric disorders, medical conditions that could interfere with cognition, that is, delirium, substance abuse, traumatic brain injury. Patients who had demonstrated early onset PD before age 45 were also excluded.

Informed consent was documented for all subjects, with accordance to the Institutional Review Board at Vanderbilt University and the ethical standards of the Declaration of Helsinki and subsequent amendments.

### Procedure

Participants were assessed with neuropsychological and UPDRS batteries pre‐ and postoperatively in their optimally treated state. Participants completed the preoperative evaluation on their regular dopaminergic medications and returned for postoperative evaluation at 6 months. With the 6‐month follow‐up, patients were on medication and had the DBS device turned on. Study data were collected using Research Electronic Data Capture (REDCap) program. REDCap is a secure, web‐based application which streamlines data gathering for research studies.

### Verbal fluency

Participants were administered the Letter and Category tasks from the DKEFS Verbal Fluency^38^ to measure Phonemic and Semantic fluency and were asked to generate action words for Action fluency.^13^ Participants were instructed to generate as many words within a minute following an action, semantic or phonemic category, such as “things people do,” “animal names,” or “words that start with the letter “f,” respectively. Participants were given points for unique individual words and were not given points for words with similar roots such as “swims” and “swimming,” nor for the same action with different subjects “riding a horse” or “riding a bike.” They were also instructed to avoid proper names of people and places, numbers. Alternate forms of these tasks were used for pre‐ and post‐DBS evaluation to reduce the confounding effects of practice for clinical purposes (i.e., patients might perform better the second time due to learning instead of beneficial DBS effects). Preoperative versions included letters F, A, S (Letter fluency) and animal names and boy’s names (Category fluency) and the postoperative version letters B, H, R and clothing and girls’ names. The order of fluency test administration was fixed across patients and consisted of Letter, Category, and Action.

### DBS contact registration

Participants considered for the study underwent a preoperative brain MRI (T1‐weighted and T2‐weighted) and a 1 month postoperative brain CT as part of standard clinical care. The MRI was acquired with a 3T Philips (Philips Achieva, Best, Netherlands) using phased‐array SENSE 8‐channel reception and body coil transmission. T1‐weighted images (typical TR/TE = 7.9/3.6 msec) were captured with 1.0 mm^3^ isotropic spatial resolution and T2‐weighted images (typical TR/TE = 3000/80 msec) with a 0.47 × 0.47 mm^2^ in‐plane resolution and 2 mm slice thickness. CT images were acquired at kVp = 120 V with 350 mAs exposure capturing 512 × 412 pixels. In‐plane resolution and slice thickness were set at approximately 0.5 mm and 0.75 mm, respectively.

The CranialVault Explorer (CRAVE) Software^39^ was used to automatically localize the implants and individual contacts in the CT images. Automatic localization was subsequently verified visually and contact position adjusted if necessary. Preoperative MRIs and postoperative CTs were registered using a fully automatic intensity‐based rigid registration techniques integrated into CRAVE. These steps allowed for visualization of individual contacts on the anatomical MRI images of the patient. The preoperative MRI was registered to the brain atlas in which deep brain anatomic structures are segmented using high field (7 Tesla) images.^40^ Registration was performed with a fully automatic intensity‐based non‐linear image registration technique (registration technique also integrated into Crave).^41^ The accuracy of the registrations was assessed visually for each volume.

This process allowed the projection of individual contacts onto the segmented atlas STN. Individual active contact positions for each patient were converted to a normalized atlas space in mm along the superior to inferior (*Y*), medial to lateral (*Z*), and anterior to posterior axes (*X*) and visualized with a web‐based interface developed at Vanderbilt University (https://www.mipresearch.org/).

### Data analysis

First, we compared the effect of stimulation in the STN and GPi on semantic, phonemic, and action fluency from pre‐ to postsurgery. Fluency for each category was defined as a scaled score (scaled score of the average word rate generated per minute). We used a mixed ANOVA with *Fluency Type* (Semantic, Phonemic, Action) and *Time* (Pre, Post) as within‐subject factors, and DBS *Target* (STN, GPi) as a between‐subject factor.

Second, to estimate which factors are associated with change in fluency from pre‐ to post‐DBS, we used a stepwise regression analyses separately for STN and GPi targets, including change in overall fluency (averaged across fluency types) as a dependent measure (change defined as: Post‐DBS – Pre‐DBS score) and normalized atlas coordinates from the active electrode position left and right superior–inferior, anterior–posterior, lateral–medial axes as independent variables.

## Results

Table 1 shows demographics, clinical variables, and DBS target effects (means and standard deviations). Table 2 reports DBS stimulation parameters for both targets (means and standard deviations).

**Table 1:** Demographics and clinical variables (means and standard deviation) for GPi and STN patients.

	GPi (17)		STN (23)		DBS target effect
	Pre	Post	Pre	Post	
Age (years)	63.03 (5.37)		63.34 (8.60)		*F* (1,39) = 0.017, *P* = 0.90
Gender (M:F)	11:6		18:5		*χ* ^2^ = 2.06, *P* = 0.15
Education (y, 15/21)	15.4 (1.8)		14.7 (2.9)		*F* (1,35) = 0.65, *P* = 0.42
Disease duration (y)	8.51 (3.36)		7.40 (3.92)		*F* (1,39) = 0.89, *P* = 0.35
LEDD	1342 (636)	1229 (625)	1444 (715)	1131 (744)	*F*pre (1,39) = 0.22, *P* = 0.64 Fpost (1,36) = 0.18, *P* = 0.67
UPDRS (on drug, off stim 17/22)	22.4 (7.9)		21.3 (9.3)		*F*pre (1,38) = 0.15, *P* = 0.70
UPDRS (on drug/on stim, 14/19)		19.9 (9.2)		18.7 (8.5)	*F*post(1,32) = 0.13, *P* = 0.72
UPDRS (off drug, off stim 16/23)	38.4 (11.7)		41.3 (9.8)		Fpre (1,38) = 0.693, *P* = 0.41
UPDRS (off drug/on stim, 14/18)		32.1 (12.02)		36.0 (14.7)	*F*post (1,31) = 0.65, *P* = 0.43
MMSE	28.53 (1.55)	28.18 (1.50)	28.96 (1.06)	28.68 (1.09)	*F*pre(1,39) = 1.11, *P* = .30 *F*post(1,39) = 1.63, *P* = 0.21
BDI‐II	16.47 (8.28)	14.88 (6.73)	12.17 (8.87)	10.26 (6.07)	*F*pre (1,39) = 2.42, *P* = 0.13 *F*post (1,34) = 4.54, *P* = 0.04

LEDD, levodopa equivalent daily dosage, BDI, Beck Depression Inventory, MMSE, Mini‐mental state examination, UPDRS, Unified Parkinson’s Disease Rating Scale.

**Table 2:** DBS clinical stimulation parameters (means and standard deviations) for GPi and STN targets separated by left and right electrode leads.

	GPi (17)		STN (23)	
	Left (17/17)	Right (14/17)	Left (23/23)	Right (21/23)
Amplitude (V)	3.1 (0.51)	3.0 (0.61)	2.2 (1.1)	2.2 (0.8)
Pulse width (msec)	74 (15)	76 (15)	72 (22)	73 (15)
Frequency (Hz)	129 (9)	128 (8)	131 (18)	129 (15)

### Analysis of sample demographics

PD patients with DBS STN and GPi targets were similar in terms of age, gender, education, disease duration, UPDRS scores, LEDD, BDI‐II, and MMSE scores. Postoperatively, the depression score was significantly higher for GPi compared to STN, although both groups show BDI‐II scores below clinically significant depression (i.e., below 20). The statistics for the comparison between targets for each demographic and clinical variables are presented in Table 1.

### DBS effect on fluency

**Figure 1:**
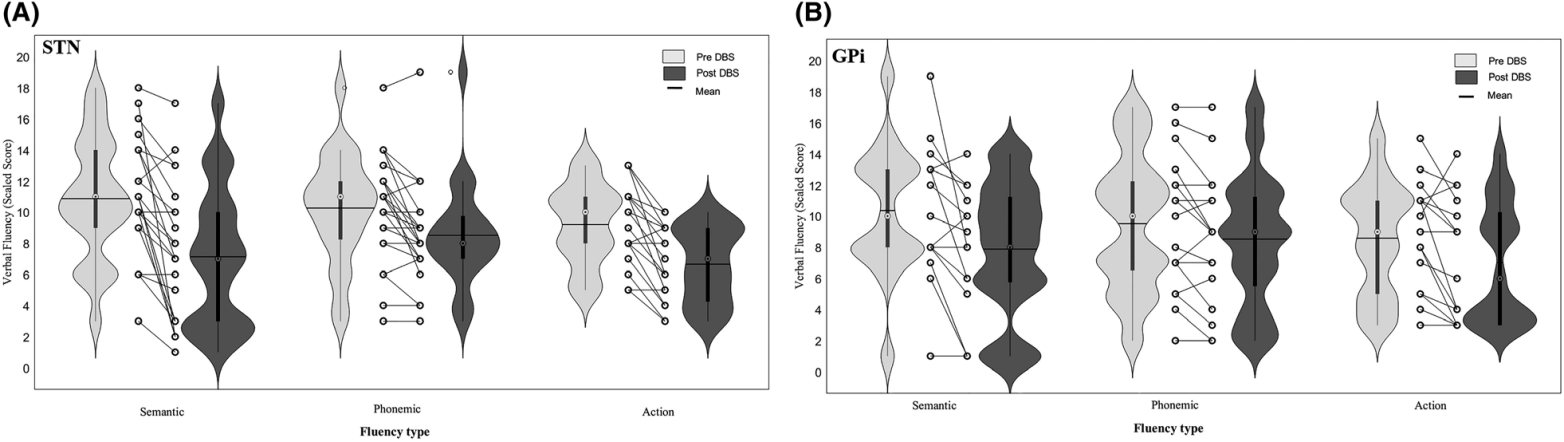
Violin box plots with mean, median, interquartile range, minimum and maximum for scaled verbal fluency scores (A) pre‐ and post‐DBS STN (B) pre‐ and post‐DBS GPi. Individual fluency changes from pre‐ to post‐DBS are displayed between the violin plots.

Figure 1A and B shows verbal fluency for each word category, pre‐ and post‐DBS surgery, separate for GPi and STN. Verbal fluency varied by word category (Fluency Type, F (2, 76) = 7.33, *P* = 0.001, *η* ^2^ = 0.16), with the production rate for phonemically associated words (9.2) and semantic‐related words (9.1) significantly higher compared to action‐related words (7.9) (phonemic‐action: *F* (1,38 ) = 15.5, *P* = 0.0003, *η* ^2^ = 0.29, semantic‐action; *F* (1,38) = 8.3, *P* = 0.006, *η* ^2^ = 0.18).

Verbal fluency decreased from pre‐ (9.8) to post‐DBS surgery (7.6) (Time, *F* (1,38) = 56.44, *P* < 0.0001, *η* ^2^ = 0.60). The postsurgical reduction in verbal fluency varied by fluency category (Time × Fluency Type, *F* (2,76) = 5.36, *P* = 0.007, *η* ^2^ = 0.12). Post hoc contrasts indicated that semantic fluency declined significantly more (from pre = 10.6 to post = 7.5) than phonemic (from pre = 9.9 to post = 8.5, *F* (1,38) = 8.07, *P* = 0.007, *η* ^2^ = 0.18) but not significantly compared to action fluency (from pre = 8.9 to post = 6.8, *F* (1,38) = 3.41, *P* = 0.07, *η* ^2^ = 0.08).

GPi and STN targets showed similar fluency declines; that is, no main or interaction effects of DBS target were found (DBS Target, *F* (1,38) = 0.02, *P* = 0.90, *η* ^2^ < 0.001; Fluency Type × DBS Target, *F* (1,38) = 0.19, *P* = 0.82, *η* ^2^ = 0.01; Time × DBS Target, *F* (1,38) = 2.93, *P* = 0.1, *η* ^2^ = 0.07; Time × DBS Target × Fluency Type, *F* (2,76) = 0.12, *P* = 0.88, *η* ^2^ = 0.003).

### Electrode position associated with postsurgery change in fluency

**Figure 2:**
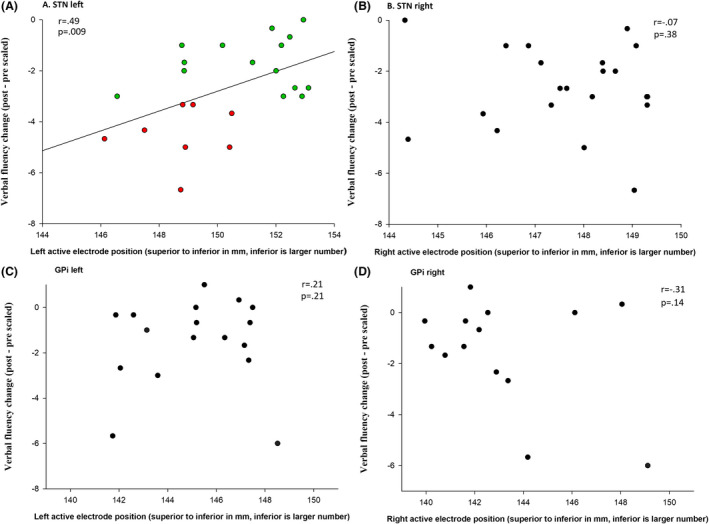
Scatterplots for (A) left and (B) right STN and (C) left and (D) right GPi correlating verbal fluency with left and right DBS electrode position depth along superior to inferior axis. Verbal fluency declines are largest when *left* active contacts are in superior STN (superior = small to inferior = large values). Red circles represent more than three points (scaled score) of clinically relevant decline in fluency, green circles less than three points of decline in fluency.

Figure 2 shows a correlation between verbal fluency decline and electrode position (left superior to inferior axis) separately for patients with GPi and STN targets.

**Figure 3:**
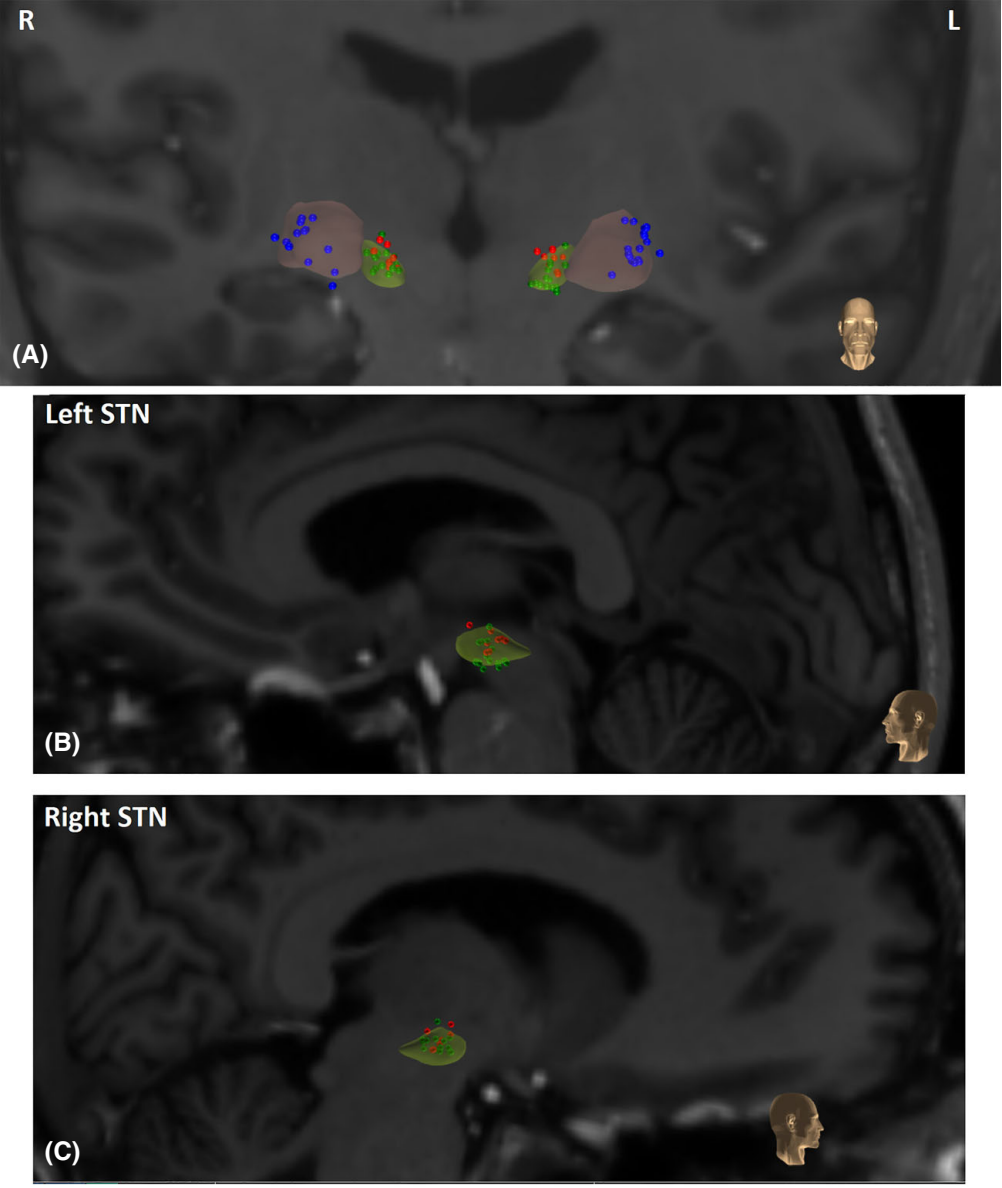
Visualization of active electrode placement in (A) STN (red and green dots) and GPi (blue dots) in normalized atlas space, coronal view. Verbal fluency decline correlated with DBS electrode position depth along superior to inferior axis in *left* STN. Red circles in STN represent more than three points of clinically relevant decline in fluency, green circles show a fluency decline of less than three points. GPi active electrode placement (blue) was not correlated with fluency change. (B) Left STN in sagittal view (C) and right STN in sagittal view.

For patients with STN targets, depth in mm on the superior to inferior axis of the left active electrode was a significant predictor of verbal fluency change (*F* (1, 18) = 5.88, *P* = 0.025, *R* ^2^ = .24). Depth of the active electrode along the left superior to inferior axis was measured in mm of normalized atlas space. Verbal fluency in patients with DBS STN improved 0.4 on a scaled score for each mm that the active contact was located toward the more inferior direction in the left STN (se Figs. 2A and 3A). Active electrode coordinates along the anterior–posterior and lateral–medial axes (left or right) were not significant predictors of verbal fluency change, rs < 0.37, ps > 0.1.

For patients with GPi targets, pre‐ to postoperative change in verbal fluency was not significantly explained by any of the electrode positions, rs < 0.45, ps > 0.08. Note that change in verbal fluency did not correlate with change in medication (LEDD) in STN (*r* = −0.05, *P* = 0.83) or GPi (*r* = 0.03, *P* = 0.93). Verbal fluency did not correlate with left or right amplitude, left or right pulse width, and left or right frequency either, GPi rs < 0.44, ps > 0.06, STN rs < 0.3, ps > 0.2.

## Discussion

The current study replicates previous findings regarding the effects of DBS on semantic, phonemic, and action fluency.^6^, ^7^, ^8^, ^33^, ^34^, ^35^, ^42^, ^43^, ^44^, ^45^ Similar to previous reports, we found postoperative fluency reductions across all fluency categories in PD patients, irrespective of stimulation site (STN or GPi). The primary goal of this study was to examine how postoperative active electrode position was associated with global verbal fluency decline. In STN‐DBS patients, the position of the *left* active electrode along the dorsal–ventral axis was associated with verbal fluency decline postoperatively. However, in GPi‐DBS patients, none of electrode positions explained the reduction of verbal fluency after DBS.

### Mechanism of verbal fluency decline

Fluency reductions after DBS have been attributed to factors related to the surgical procedure (e.g., lesion effects, trajectory impact) as well as to stimulation parameters.^21^, ^23^, ^25^, ^43^ Our particular focus was to understand the roles of the clinically determined active electrode positions within each hemisphere to fluency decline. Specifically, we investigated how the variations of electrode positions within and across patients were associated with changes in verbal fluency from presurgical testing to 6 months after DBS implantation. The current study suggests that in patients with bilateral STN implants, the position of the *left* active electrode was associated with verbal fluency decline postoperatively, similar to fluency reductions with left DBS reported in previous stimulation studies with unilateral STN, and in line with a left‐lateralized specialization of the brain for verbal fluency.^25^, ^28^, ^46^, ^47^, ^48^, ^49^, ^50^

The STN is thought to contain separate functional subregions and stimulation of the dorsolateral motor region generally results in the best motor outcome.^51^, ^52^ Spread of DBS stimulation to more medial associative and ventral limbic regions has been linked to various cognitive and emotional regulatory side effects. In fact, it has been suggested that stimulating at a relatively more ventral contact in the STN may contribute to fluency decline.^34^ However, our findings indicate that the largest fluency declines occurred in patients whose clinically active contacts were positioned in a relatively more dorsal subregion of the left STN.

How could we explain the apparent discrepancies in the findings? One possibility is that the effect of DBS on fluency depends on the specific tracts within the corticostriatal network that are stimulated rather than on the exact region of the active electrode within STN, and this is currently challenging to compare between studies. For example, the superior (dorsal) active contacts in our study could have activated a slightly different cortical–striatal network compared to the dorsal contacts stimulated in, other studies.^30^, ^34^ Recent technological advancements combining diffusion tensor imaging (DTI) coupled with VTA could aid our understanding of which fiber tracts are modulated by the VTA and are related to stimulation (versus lesion)‐induced fluency decline in GPi and STN.^53^, ^54^ Alternatively, white matter pathways could be directly or indirectly affected by DBS. For example, Costentin et al.^55^ recently reported surgically induced microlesions of the frontal aslant fascilus, a frontal white matter pathway connecting the inferior frontal gyrus (IFG) to presupplementary motor area (pre‐SMA), with ties to verbal fluency,^56^ although they did not find a direct association with the verbal fluency decline post‐DBS. Notably, the IFG and pre‐SMA project directly to mid‐dorsal regions of the STN.^52^ Future studies should consider how altered communication between these cortical areas due to microlesions vis‐à‐vis altered communication due to STN stimulation contribute to verbal fluency changes.

In the current study, fluency declined with stimulation in the STN as well as in the GPi, although active electrode position did not explain fluency changes in GPi. The GPi is a larger structure and the electrode lead is generally entirely positioned in the motor region, thus nonmotor regions are less likely to be affected.^29^, ^31^ The current results suggest that stimulating in the motor territory of the GPi produces a similar effect on fluency as stimulating in the relatively more dorsal, motor territory of the STN. One possibility is that stimulating motor regions in GPi and in the dorsal subregion of the STN modulates cortical–striatal circuits including presupplementary motor areas (pre‐SMA) and dorsolateral prefrontal cortex (DLPFC), brain areas involved in the executive control of verbal fluency.^57^ For example, reductions in fluency with bilateral DBS have been associated with diminished activity in the left dorsolateral prefrontal cortex and left Broca’s area.^17^ Reductions in fluency with DBS in motor regions (of STN or GPi) may be partially driven by disruptions to executive control processes that are involved in verbal fluency like switching, inhibition, and selection.^7^, ^8^, ^19^, ^27^, ^33^ Future research using microelectrode neurophysiological recordings in cortical and subcortical areas during verbal fluency performance could aid in clarifying the role of the STN and GPi in verbal fluency.

### Limitations and future directions

This study has several limitations. First, although we were able to take advantage of the within‐subject nature of our sample, the smaller samples sizes in each group (~20 per group) limits the breadth of our conclusions. While the results reveal statistical support that electrode positions along the dorsal–ventral axis in the left STN are associated with fluency decline, future studies with larger samples are needed to confirm the robustness and strength of these relationships. A larger sample will provide more statistical power to investigate a combination of stimulation parameters in both DBS targets, like voltage, frequency and pulse width, volume of tissue activation, and surgical lesion factors such as lead trajectory.

Second, the current study is limited because it was a retrospective study that did not allow full experimental control over the variables and this could have impacted our findings. A future prospective study with for example a randomization of the DBS targets across patients, an equal number of participants with unilateral versus bilateral targets, and standardized stimulation parameters would overcome some of these confounds.

Moreover, we studied patients in their optimal medication and stimulation state and did not include a postoperative off stimulation state to dissociate potential surgical lesion from stimulation effects. Two recent studies that investigated the stimulation effect on verbal fluency, by comparing ON‐OFF stimulation performance, did not find a DBS effect.^58^, ^59^ Similarly, a review on DBS‐induced cognitive decline^14^ suggested that verbal fluency appears to be largely a surgical implantation effect. Others have pointed out the impact of stimulation parameters and contact location as contributing factors, especially in terms of individual variation in fluency effects.^21^, ^34^, ^59^ Finally, the limited number of unilateral patients in the current study did not allow us to dissociate the contribution of unilateral versus bilateral stimulation on fluency changes; this should be addressed in future studies.

However, running a fully factorial design is quite challenging, given issues related to unilateral versus bilateral implantations, quantifying contact locations, medication states, and the demanding designs required to test stimulation effects across hemispheres, contacts, and related stimulation parameters. The current study contributes another piece of the puzzle to our understanding of stimulation factors related to verbal fluency declines with DBS.

## Conclusion

The current study shows a clear reduction in verbal fluency 6 months after surgical implantation of electrodes and stimulation targeting either STN or GPi targets in patients with PD. When DBS targets the STN, fluency declines were associated with clinically active electrodes positioned along the dorsal–ventral axis. Clinically, including a fluency measure during the process of defining and optimizing the DBS parameters could maximize both motor and fluency outcomes.

## Conflict of Interest

Dawant is a founder and equity holder in Neurotargeting, LLC., that licenses some of the technology from Vanderbilt University described in this article. Phibbs has done consulting for Boston Scientific, Medtronic and Teva. There is no other conflict of interest to report.
